# Traumatic Dental Injuries: Prevalence, First Aid, and Mouthguard Use in a Sample of Italian Kickboxing Athletes

**DOI:** 10.3390/dj12100310

**Published:** 2024-09-29

**Authors:** Roberto Biagi, Cristina Mirelli, Rebecca Ventimiglia, Saverio Ceraulo

**Affiliations:** 1 Department of Biomedical, Surgical and Dental Sciences, School of Dentistry, University of Milan, 20122 Milan, Italy; cristina.mirelli@unimi.it (C.M.); dott.ssa.rebeccaventimiglia@gmail.com (R.V.); 2 Fondazione IRCCS Cà Granda Ospedale Maggiore Policlinico, UOC di Chirurgia Maxillo-Facciale e Odontostomatologia, Via della Commenda 10, 20122 Milan, Italy; 3 Department of Medicine and Surgery, University of Milano-Bicocca, 20900 Monza, Italy; saverio.ceraulo@unimib.it; 4 Fondazione IRCCS San Gerardo dei Tintori, Via Pergolesi 33, 20090 Monza, Italy

**Keywords:** sports, martial arts, kickboxing, traumatic dental injuries, tooth avulsion, mouthguard

## Abstract

Background: Traumatic dental injuries (TDIs) are a common complication in sports, with an overall prevalence ranging from 0.71% to 60%. Kickboxing is a high-risk combat sport for trauma to the facial region. Methods: A total of 142 5-to-65-year-old amateur athletes participated in the survey with a questionnaire that was released during their regular practice days. The questionnaire was structured into three parts: (1) questions about age, gender, type, and time of sports practice; (2) questions about dental injuries, personal experience, and awareness of emergency management, particularly about tooth avulsion; (3) questions about wearing a mouthguard for TDI prevention. Results: A total of 13 percent of athletes suffered a TDI while training or fighting. Regarding first aid, 61% of the participants in this survey are aware of the possibility of replanting a tooth after its avulsion; 10% believe that the tooth should be replanted within 15 min, and 37% of athletes believe that it should be kept wet. The mouthguard is known to the majority of athletes (94%), and 66% were informed by the coach; 68% of the athletes use it. Seventy-four percent of the mouthguards were purchased in a sports shop. Conclusions: A relatively low prevalence of TDIs was observed in players in the sample. A lack of information about the emergency management of tooth avulsion and about the type of mouthguard to wear emphasizes the importance of educational training by dentists to athletes and coaches.

## 1. Introduction

Traumatic dental injuries (TDIs) can be considered the fifth condition of the world’s acute/chronic diseases and injuries [1] and represent a global health problem. They are a common complication in sports activities, with an overall prevalence ranging from 0.71% to 60% [2]. Federation Dentaire International (FDI) classifies sports such as martial arts as a high-risk sport because rough contact between the players is allowed [3]. Kickboxing is a martial art in which punching, kicking, and, under some rules, kneeing and elbowing are permitted. It is classified as high risk for dentofacial injuries due to the body movement that can reach the facial region; therefore, the use of a mouthguard is recommended to reduce the severity of trauma and dental treatment needs, and it is mandatory during competitions. The aim of this cross-sectional study is to evaluate the prevalence of TDIs during kickboxing practice, emergency management, particularly when a tooth avulsion occurs, and finally, the knowledge and use of a mouthguard.

## 2. Materials and Methods

A questionare (Table 1), similar to those used in previous surveys [4,5,6,7], investigating the past history of dentofacial trauma (lifetime prevalence) was released during regular practice days to 161 athletes aged 5–65 registered to Associazione Sportiva Dilettantistica D.F.K.T. Fight Team Indomita in Sanremo, a town in Northern Italy. Professional and semi-professional athletes were not included in this study because the members of this sports club were only amateurs. Kickboxing was the only sport considered.

The questionnaire was structured into three parts: (1) questions about age, gender, type, and time of sports practice; (2) questions about dental injuries, personal experience, and awareness of emergency management, particularly about tooth avulsion; (3) questions about TDIs prevention wearing a mouthguard. The meaning of each question was clearly explained before completing the questionnaire, and informed consent obtained from parents of under-aged athletes or individual adult participants was obtained.

The limit of this cross-sectional study is that the sample consists exclusively of amateur athletes.

## 3. Results

### 3.1. Sample of the Survey

**Figure 1:**
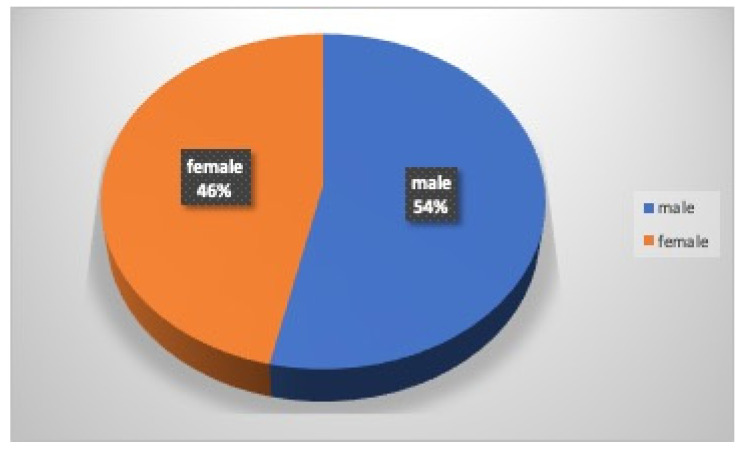
Gender of the athletes.

**Figure 2:**
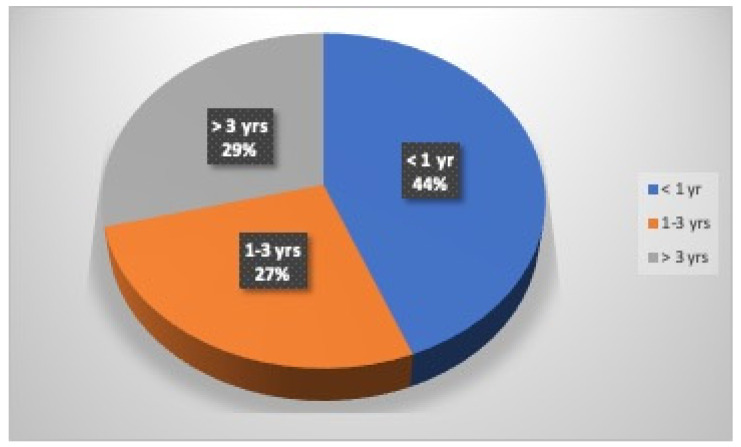
Kickboxing practice.

Of a total of 161 athletes who received the questionnaire, 142 (88%) returned it: 76 (54%) were males, and 66 (46%) were females (Figure 1). Thirty-eight (27%) athletes were under 18 years old: 23 (61%) were males, and 15 (39%) were females; 104 (73%) athletes were over 18 years old: 53 (51%) were males, and 51 (49%) were females. Forty-four percent of the athletes had been practicing kickboxing for less than one year (30% males and 59% females), 27% from one to three years (25% males and 30% females), and finally, 29% more than three years (45% males and 11% females) (Figure 2). Only 24% of the athletes (33% males and 13% females) played this sport at a competitive level.

### 3.2. TDIs

**Figure 3:**
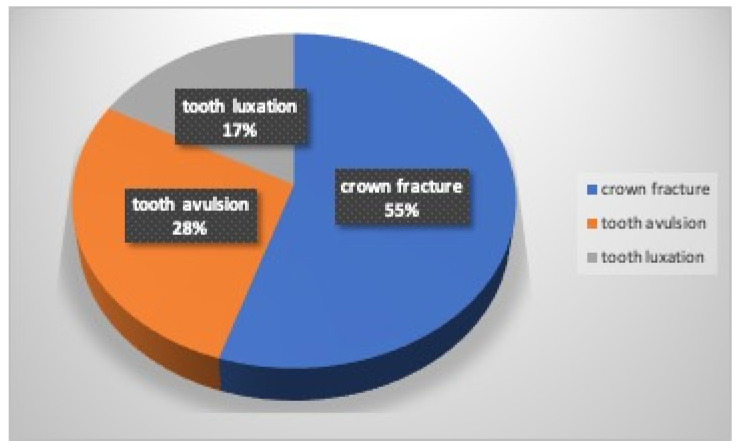
Kind of dental injury.

In all, 18 athletes (13%) reported that they suffered a dental trauma while training or fighting in equal measure (nine males and nine females). The kind of dental injury they had was 55% crown fracture (67% males and 45% females), 28% tooth avulsion (22% males and 33% females), and 17% tooth luxation (11% males and 22% females) (Figure 3).

### 3.3. First Aid

**Figure 4:**
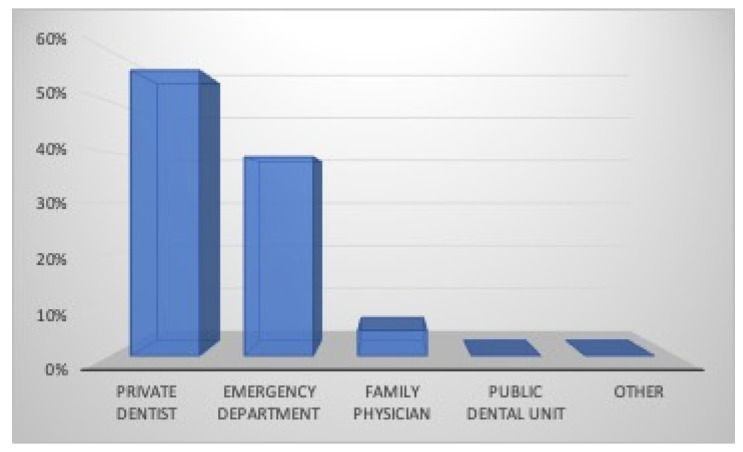
Emergency management.

**Figure 5:**
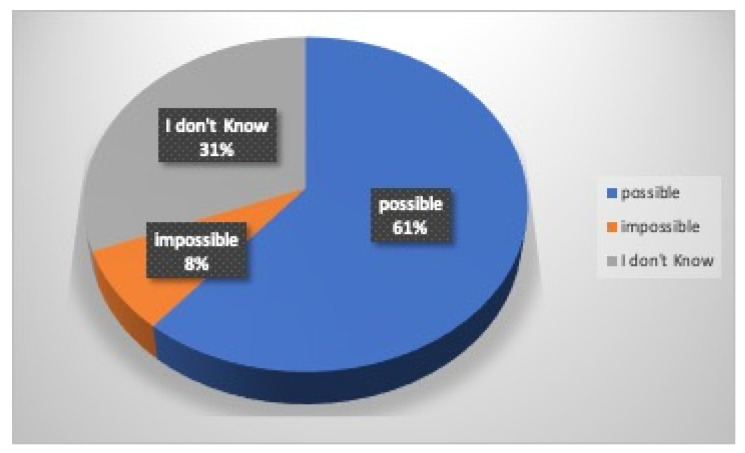
Replantation of a tooth.

**Figure 6:**
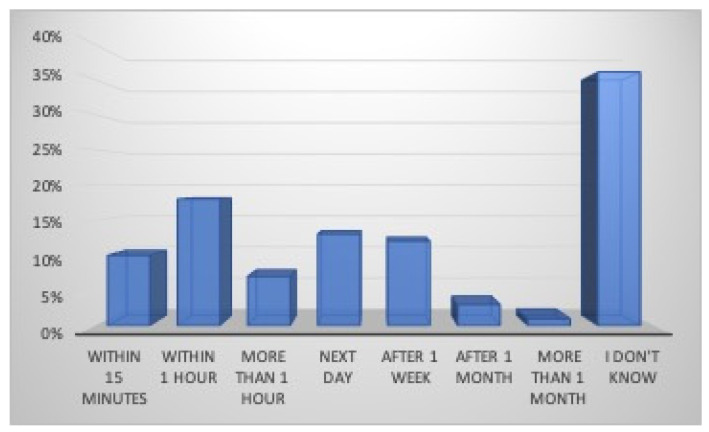
Preferable time to replant a tooth.

**Figure 7:**
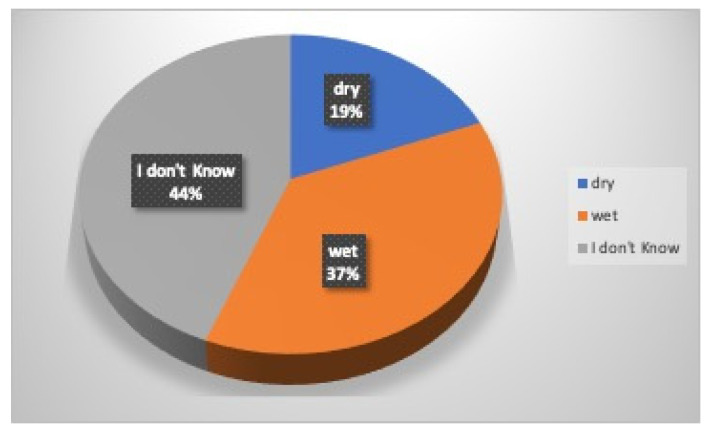
Storage of a tooth.

Regarding first aid, 56% of the athletes (70% males and 38% females) turned to their private dental practitioner, 39% (20% males and 62% females) to a Hospital Emergency Department, 5% to their family physician or pediatrician (only males), while no one turned to a Public Dental Unit (Figure 4). Eighty-five percent of the athletes believe in the importance of immediate professional management of dental injury in order to increase the rate of success, while 4% think it does not matter, and 11% do not know; 61% of the participants in this survey know the possibility of replanting a tooth after its avulsion, while 8% think that this is impossible and 31% do not know (Figure 5). According to the participants who would reimplant the avulsed tooth in its socket, 10% (7% males and 14% females) believe that the tooth should be replanted within 15 min, 18% (44% males and 27% females) within an hour, 7% more than an hour, 13% the next day, 12% after a week, 3% after a month, 1% more than a month, and 36% do not know (Figure 6). Nineteen percent of athletes think that the tooth should be kept dry (14% males and 25% females), 37% think that it should be kept wet (30% males and 43% females), while 44% (56% were males and 32% females) do not know how to store it (Figure 7).

### 3.4. Mouthguard

**Figure 8:**
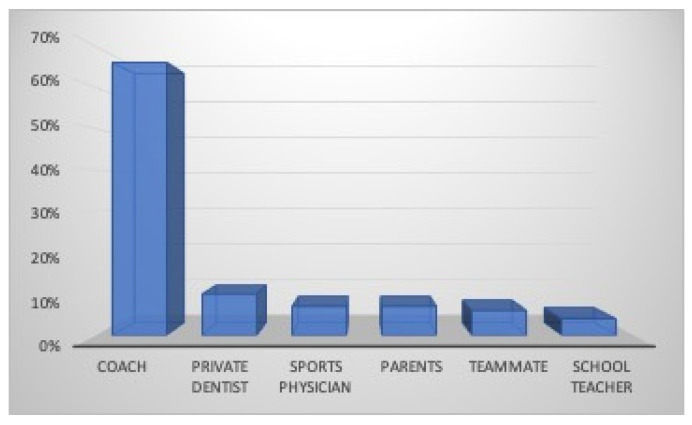
Athletes’ information about mouthguard use.

**Figure 9:**
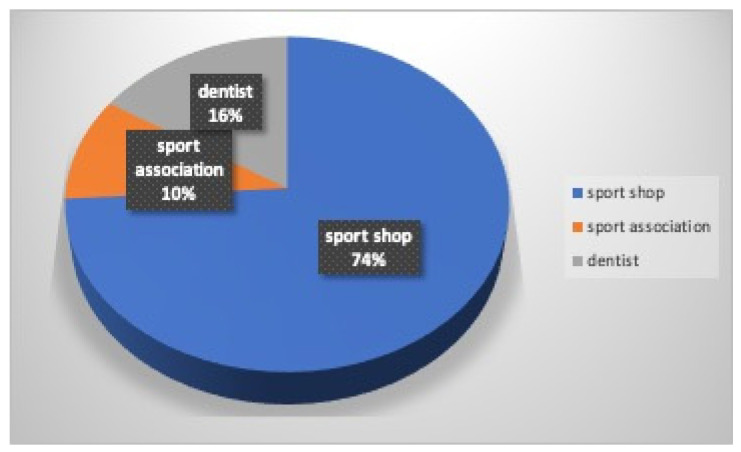
Mouthguard providers.

**Figure 10:**
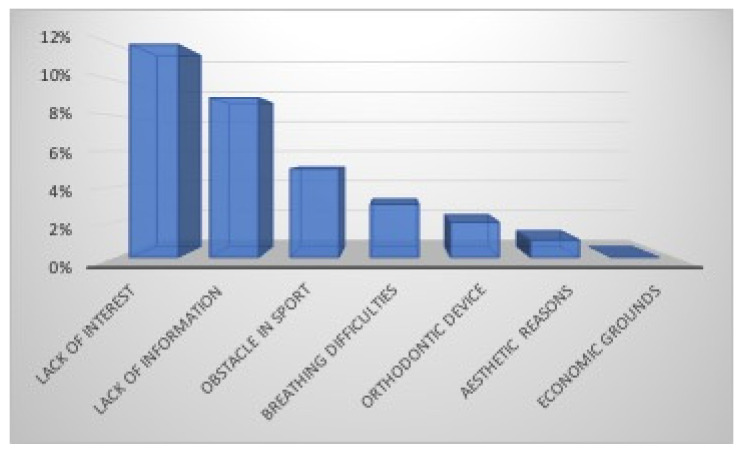
Lack of mouthguard use.

The mouthguard is known by the majority of athletes (94%), and they were informed by the coach (66%), their private dental practitioner (10%), the sports physician (7%), the parents (7%), a teammate (6%), and the school teacher (4%) (Figure 8). The mouthguard is used by most players (68%: 74% males and 61% females) during sports activities. The mouthguard was purchased in a sports shop (74%) or provided by the Sports Association (10%,) while it was supplied by the dentists to 16% of the athletes (Figure 9). The main reason for its lack of use by 32% of athletes was due to lack of interest (12%) or information (9%), while 11% believed that it represented an obstacle in sport (5%), created breathing difficulties (3%), for an orthodontic treatment in progress(2%), or refused wearing it for aesthetic reasons (1%). Nobody justified their decision on economic grounds (Figure 10).

### 3.5. Athletes’ Interest for the Survey

Most athletes (86%) found it interesting and useful to participate in this study, while only 5% expressed a negative opinion. Nine percent were already aware of mouthguards and emergency management of dental trauma.

## 4. Discussion

Dental and facial injuries are highly correlated with sports activities, with an overall prevalence ranging from 0.71% to 60% [2]. This variability of rates depends on the type of sport, selected group of athletes, geographical location, age of the athlete, sample size, level of competition, data collected from coaches, emergency departments, or dental clinics, and finally on the use of mouthguards or other protective equipment [8]. Sports categories are divided into non-contact sports and contact sports. Martial arts are considered contact sports due to the continuous physical interaction. Kickboxing, a martial art that permits punching, kicking, and, under some rules, kneeing and elbowing, is classified as high risk for TDIs for this reason. Unlike other surveys, which report data related to more than one of the most practiced martial arts, such as jiu-jitsu, judo, karate, kendo, kickboxing, kung fu, Muay Thai, and taekwondo, this survey considers only kickboxing.

### 4.1. TDIs

This study analyzes the data obtained from 142 questionnaires that were distributed by a dentist to amateur athletes aged 5–65 during their training: only 24% of the athletes played this sport at a competitive level. Eighteen athletes (13%) reported that they suffered dental trauma while training or fighting. The kind of dental injuries they had were as follows: 10 (55%) crown fractures; 5 (28%) tooth avulsions; and 3 (17%) tooth luxations. A percentage of 11.38%, similar to that reported in this study, was found in contact sports [2]. However, if we consider kickboxing alone, it has the highest incidence of dental trauma. Within a group of 120 athletes practicing four contact sports (boxing, taekwondo, kickboxing, and Muay Thai), 53 athletes (44.2%) suffered a TDI, and 66.7% of them were due to kickboxing; tooth fractures (59.7%) were the most common type observed [9]. Horri et al. [10] report a prevalence of dental trauma of 37.8% (*n* = 133) in a sample of 352 athletes practicing six different combat sports (boxing, karate, kickboxing, kung fu, taekwondo, and wushu). Twenty-eight athletes (21.1%) suffered dental trauma, and tooth luxations were the most frequent injuries (13.2%, *n* = 43); tooth fractures and tooth avulsions occurred in 9.7% (*n* = 34) and 5.7% (*n* = 30), respectively. The lower prevalence of dental trauma observed in the present survey may be due to the fact that kickboxing is practiced at an amateur level (semi-professional and professional athletes were not included in the sample), and only 24% of the athletes played this sport at a competitive level. Due to the heterogeneity of the data presented in the literature, a comparative evaluation of the prevailing type of dental trauma is difficult, although dental fractures were observed with prevalence in combat sports [11].

### 4.2. First Aid

International Association of Dental Traumatology (IADT) guidelines emphasize that the immediate management of most TDIs is essential for the long-term survival of traumatized teeth [12,13,14,15]. Eighty-five percent of enrolled kickboxing athletes felt that immediate management of dental injuries by a dentist was very important to increase the success rate, and this percentage was higher than that of another similar survey [5]. The TDI was managed by a private dentist in 56% of the players. Thirty-nine percent of the injured athletes are referred to the Hospital Emergency Department, where a dentist is rarely a team member in Italy; therefore, it is inevitable that if medical physicians have not received prior training in the management of TDIs, they are unable to provide an appropriate emergency treatment of dental trauma [16,17]. Guidelines of IADT should assist in decision-making in a Hospital Emergency Department, and their application could maximize the chance of a favorable outcome [12,13,14,15]. The patient is usually sent to a dentist for emergency management with an inevitable delay, to the patient’s detriment. Dental avulsion is one of the most serious dental traumas, and for this reason, it was investigated in the present survey. Fortunately, only five athletes have suffered this injury. Sixty-one percent of the 142 athletes believe that a tooth can be replanted, preferably within 15 min, according to 10% of the athletes, and kept in wet storage, according to 37% of them, respectively. Immediate replantation of the avulsed tooth at the place of the accident is the best practice even if the tooth replanted within 15 min after the avulsion has a favorable long-term prognosis [18]; but if for some reasons, such as life-threatening traumatic injuries, complex injury to the alveolar socket, or lack of awareness about immediate replantation, this is not feasible, the tooth must be kept in a wet physiologic storage medium to preserve the viability, mitogenicity, and clonogenic capacity of periodontal ligament (PDL) cells [14]. It is, therefore, very important to select an optimal medium to facilitate the healing and to improve the survival rate of the reimplanted tooth. To date, many storage media (Hank’s balanced salt solution, aloe vera gel, oral rehydration solution, coconut water, egg white, green tea, propolis, saline, milk, water, etc.) have been used in clinical practice. Hank’s Balanced Salt Solution (HBBS) is one of the ideal media [19,20,21,22]. HBBS has a pH balanced at 7.2, an osmolarity of 320 mOsm/kg, and preserves viability, mitogenicity, and clonogenic capacities of PDL cells for up to 24 h. Lee et al. [23] compared two commercial storage media to HBBS: Save-A-Tooth (SAT) and EMT Toothsaver (EMT). SAT (Phoenix-Lazerus, Inc., Shartlesville, PA, USA) is an HBBS-based solution, while EMT (SmartPractice.com, Phoenix, AZ, USA) is made of Special Cell Culture Medium, which is a modified version of Roswell Park Memorial Institute medium that contains a combination of amino acids, vitamins, and glucose. EMT is marked as Dentosafe (MEDICE Arzneimittel Pütter GmbH & Co. KG, Iserlohn, Germany) in Europe. No significant difference between HBSS, SAT, and EMT was found for up to 6 h, while SAT was effective up to 12 h, and only EMT retained its effectiveness after 24 h. Unfortunately, these media are very rarely available at the site of the accident. Thus, it is recommended to distribute Dentosafe at locations where tooth traumas can easily occur (schools, kindergartens, sporting facilities, public pools) and at emergency units (hospitals, ambulances) to enhance the prognosis of avulsed teeth. Based not only on PDL cell viability but also on practical considerations such as high availability and low cost, milk is one of the most commonly used and recommended storage media. Milk has a physiologic pH of 6.5–7.2 and an osmolarity of 270 mOsm/Kg. Pasteurized and refrigerated fresh milk with lower fat is preferable. The PDL cells have been shown to survive for 2–6 h when immersed in milk, and it is effective as HBBS for up to 1 h [19,24,25]. Saliva can be recommended as an immediate temporary storage medium until a better storage medium, such as milk, is found near the site of the accident. Saliva has a pH that can range from 5.3 to 7.8 and an osmolarity of 60–70 mOsm/Kg, lower than a physiological one: this feature causes cell swelling, which stretches the cell membrane and potentiates the effect of bacterial products and toxins present in it. In order to avoid the risk of aspiration or swallowing the avulsed tooth, saliva or a saliva-and-blood mixture spat or drooled into a receptacle may be preferred. Saliva may serve as a storage medium for up to 2 h, but currently, a storage time no longer than 30 min is recommended [26,27,28]. First aid may be handled by everyone, so the knowledge of emergency procedures by athletes and coaches is very important.

### 4.3. Mouthguard

Mouthguards are considered the main appliance for the prevention and reduction in severity of sports-related dental injuries [29,30,31], and they are known by 94% of the athletes in this survey, with a percentage higher than that detected by Biagi et al. [5], Ferrari and Medeiros [32], Aliobani et al. [33], Guinot and Manrique [34] and lower than that detected by Vidovic et al. [6] and Galic et al. [7]. Sixty-eight percent of kickboxing athletes used mouthguards, with a percentage comparable to that reported by Aliobani et al. [33], while even though 81.4% were aware of mouthguards, only 19.86% of jiu-jitsu athletes used them, according to the results published by Ferrari and Medeiros [32].

Only 66% of kickboxing practitioners were informed by their coaches about the use of mouthguards. The coaches can play a positive role in raising awareness and in the use of mouthguards because compliance is lower when proper education is not provided. Three main types of mouthguards are available: (a) pre-fabricated, that are pre-made in different sizes, not adaptable, and the patients choose the best-fitting one; (b) mouth-formed, commonly referred to as “boil and bite”, which is made from thermoplastic material, adaptable to the patient’s teeth, soft tissues, and occlusion after having been heated in hot water; (c) custom-made, that are fabricated by dental laboratories from dental impressions using polyethylene vinyl acetate (EVA). The dentist checks the fit of the mouthguard [30,31]. In this survey, most mouthguards (74%) were purchased at a sports shop; only to a lesser extent were they provided by the Sports Society (10%) or supplied by the dentists (16%), and the “boil and bite” mouthguard was the most used type mainly for their easy availability at affordable prices in sports stores and lack of knowledge about the custom-made ones. Due to the continuous oral changes in mixed dentition age, they are also preferred in children for their possibility of remolding [35] and during fixed orthodontic appliance treatment for the same reason, but it is important to emphasize two aspects: length and thickness. An insufficient length and thickness mean lower protection. Custom-made mouthguards offer a superior fit and protection compared to the other types of mouthguards [30,31], and they can be designed to incorporate a cut out channel for the orthodontic appliance. In this study, the main reasons for not using a mouthguard were represented by the lack of interest (12%) and information (9%) by athletes; 2% also believed that it could not be used for orthodontic treatment in progress, and 1% did not wear them for aesthetic reasons. A total of 5% justified their decision because they believed that the mouthguard hindered their sports performance, and according to 3%, it created breathing difficulties. Compared with 32% of athletes in this survey who did not use a mouthguard, other studies report higher percentages in contact sports [5,32,33,34,35], but the mouthguard is mandatory in kickboxing. Discomfort and difficulty breathing were also reported by athletes participating in other investigations [5,10,33,34,35] and are due to the constant occlusal pressure necessary to hold mouthguards in place for lack of proper retention. Keçeci et al. [36] evaluated that custom-made mouthguards do not significantly affect the ventilatory gas exchange of taekwondo elite athletes while performing maximal exercise, providing them with greater comfort and safety.

## 5. Conclusions

The results of this study show a relatively low prevalence of TDIs in kickboxing athletes. Most players in the sample lacked information on first aid for tooth avulsions. Nearly all acknowledged the importance of a mouthguard to reduce the number and the severity of TDIs, but many of them did not know what type to wear. Future research will be very useful in obtaining further data, particularly on combat sports-related dental injuries, in order to emphasize the importance of educational training regarding emergency management of TDIs and the use of custom-made mouthguards for athletes and coaches. Posters and contemporary means like the internet and apps can be a valuable help.

## Figures and Tables

**Table 1:** Questionnaire.

How long have you been practicing kickboxing? (less than 1 year, 1–3 years, more than 3 years)
Do you practice kickboxing at a competitive level?
Have you ever suffered a dental injury?
If yes, what kind of injury? (crown fracture, avulsion, dislocation)
Where would you go for first aid? (private dental practitioner, Hospital Emergency Department, family physician or pediatrician, Public Dental Unit, other: please specify)
Do you think that immediate management of dental injuries by a dentist is important to increase the rate of success? (yes, no, I do not know)
Do you think it is possible to replant a knocked-out tooth? (yes, no, I do not know)
If yes, within which time frame is it preferable to replant the tooth? (15 min, within 1 h, more than 1 h, 1 day, 1 week, 1 month, more than 1 month, I do not know)
If yes, what is the right way to store it? (dry, wet, I do not know)
Do you know what a mouthguard is?
Who told you about this? (coach, private dental practitioner, sports physician, parents, teammate, schoolteacher)
If yes, how did you buy it? (sports shop, Sports Association, dentist)
Do you wear a mouthguard?
If not, why? (lack of interest, lack of information, obstacle in sport, breathing difficulties, presence of orthodontic device, economic grounds)

## Data Availability

The data presented in this study are available in this article.
